# Research priorities in infertility and assisted reproductive technology treatments - a James Lind Alliance priority setting partnership with brazilian patients

**DOI:** 10.5935/1518-0557.20190077

**Published:** 2020

**Authors:** Aline R. Lorenzon, Désirée Garcia, Leticia Silva, Cristiane Araújo de Oliveira, Mauricio B. Chehin, Ricardo Mello Marinho, João Pedro Junqueira Caetano, Rita Vassena, Eduardo Leme Alves da Motta

**Affiliations:** 1 Huntington Medicina Reprodutiva, São Paulo, SP, Brazil; 2 Clinica Eugin, Barcelona, Spain; 3 Pró-Criar Medicina Reprodutiva, Belo Horizonte, MG, Brazil; 4 Faculdade Ciências Médicas Minas Gerais, Belo Horizonte, MG, Brazil; 5 Departamento de Ginecologia, Escola Paulista de Medicina, Universidade Federal de São Paulo, São Paulo, SP, Brazil

**Keywords:** James Lind Alliance Priority Partnership, infertility, Brazilian patients, *in vitro* fertilization

## Abstract

**Objective:**

To identify the main research interests of Brazilian patients in the field of infertility and assisted reproductive technology (ART) treatments.

**Methods:**

This prospective multicenter cross-sectional study was carried out in Brazil. Patients attending five fertility centers from the Huntington Group between October and December 2018 were invited to join the study, which consisted of answering an anonymous survey online. Two hundred and twenty-seven patients signed the informed consent form and were emailed the survey link. The survey was designed based on the James Lind Alliance Priority Setting Partnership protocol. In the area of infertility, patients were probed on issues such as somatic and psychological effects of treatment, prevention, assisted reproductive technology (medications and procedures), success rates, risks, and emotional aspects.

**Results:**

The response rate (RR) was 47.58% (108 patients; 88 women - RR 51.46% and 20 men - RR 35.71%). Patient mean age was 36.5 years (SD 4.6). The top ten research priorities listed were 1) short- and long-term side effects of treatment; 2) how to cope with infertility; 3) risks associated with ART; 4) success rates in ART; 5) impact of diet on ART and fertility; 6) healthy habits; 7) alternative therapies; 8) impact of exercise on fertility and ART success; 9) oocyte quality and ovarian reserve; and 10) genetic or inherited causes of infertility.

**Conclusion:**

To better cater to the needs of patients and develop patient-centered care in the field of infertility and ART treatment, clinicians, healthcare providers, and the scientific community must identify patient concerns and priorities and make efforts to address them.

## INTRODUCTION

Infertility - a condition affecting over 50 million people worldwide and approximately 8 million couples in Brazil (IBGE, 2010) - is the inability of a sexually active, non-contracepting couple, to achieve pregnancy in one year (WHO, 2019). Despite global research on the development new drugs, techniques, and devices for assisted reproduction technology (ART) procedures to improve therapy success rates, the diagnosis and clinical treatments prescribed to treat infertility and reproductive disorders usually lead to emotional distress and anxiety in patients (Gdańska *et al.,* 2017).

The last decade saw an increase in the involvement of patients in the definition of research topic priorities in fields in which they participate as the subjects of interest. There is a growing concern that research funds have been spent to cater to the interests of healthcare providers, caregivers, or the academia rather than to the needs of patients (JLA, 2019). A mismatch between what is considered a research priority for patients or healthcare providers is common in many fields of medicine (Crowe *et al.,* 2015).

The James Lind Alliance (JLA) is a National Institute for Health Research-supported initiative in the United Kingdom that aims to bring patients, carers, and clinicians together to identify and prioritize the top unanswered questions that they agree are the most relevant in a medicine field (JLA, 2019). It also aims to ensure health research funders are aware of the topics that are most relevant for both patients and researchers. To achieve this goal, the JLA developed a set of guidelines for the Priority Setting Partnerships (PSPs) model, which consists of four main phases: 1) identification of patient groups - exploration; 2) investigation of the research agenda through surveys, interviews, and/or literature reviews - consultation and prioritization; 3) data collection, analysis, and identification of priorities - integration; 4) evaluate how the patient perspective can be included to build a research agenda - incorporation. 

The JLA PSPs model has been explored in many areas of medicine, including diabetes types 1 and 2 (Gadsby *et al.,* 2012; Finer *et al.,* 2018), Parkinson’s disease (Deane *et al.,* 2014), and some types of malignant disease such as kidney (Jones *et al.,* 2017) and prostate cancer (Lophatananon *et al.,* 2011). Some elements of the PSPs have been applied in reproductive medicine, in areas such as endometriosis (Horne *et al.,* 2017); in gynecology, in cases of endometrial cancer (Wan *et al.,* 2016); and in obstetrics to look into gestational diabetes (Rees *et al.,* 2017), miscarriage (Prior *et al.,* 2017), stillbirth (Heazell *et al.,* 2015), and preterm birth (Duley *et al.,* 2014).

Postponement of childbearing and aging-related declines in gamete biological potential are listed as the top reasons for the increased use of infertility and ART treatments (Ubaldi *et al.,* 2019; Jennings *et al.,* 2017). The JLA PSPs model has not been applied to infertility or ART treatments yet. Here, we developed a survey modeled after the JLA PSPs in which Brazilian patients prioritized the top ten research questions in the field of infertility and assisted reproductive technology treatment.

## MATERIALS AND METHODS

This prospective multicenter cross-sectional study included patients attending five fertility centers for either appointments or clinical ART procedures between October and December 2018. The included individuals were invited to join the study, which consisted of anonymously answering an online survey. After signing an informed consent form, participants were emailed a link from which they opened the survey questionnaire. The Ethics Committee of the Federal University of São Paulo (UNIFESP) approved the study (certificate no.: 92116918.5.0000.5505).

### The survey

Clinica Eugin Barcelona, Spain, designed the survey according to the JLA PSPs model (DG and RV) and tested it in a pilot study with 100 randomly selected patients attending their first appointments in 2017. After validation of topics and categorization, the survey was applied in our study.

The survey was designed in an online platform (Google Forms), and took approximately 20 minutes to answer. In the area of infertility, patients were probed on issues such as somatic and psychological effects of treatment (used to describe the group of patients, not to define research priorities), prevention, assisted reproductive technology (medications and procedures), success rates, risks, and emotional aspects. The survey was designed based on other studies on the JLA PSPs (van Middendorp *et al.,* 2016; Deane *et al.,* 2014) and is available in Annex 1.

### Data analysis

Survey answers were analyzed and categorized by two authors according to the topics validated during the pilot study and based on JLA methodology. We extracted the frequency of each topic and the aggregated categorized answers and identified a long list containing the top thirty main research interest areas and a shortlist of the top ten research interests in infertility, all based on patient answers. Closely related categories were grouped in an effort to select the top ten research interests, such as general and long-term side effects.

## RESULTS

### Population

Two hundred and twenty-seven patients were prospectively included in this study, consisting of 171 women (75.33%) and 56 men (24.67%). The overall response rate (RR) was 47.58% (108 patients in total; 88 women - RR 51.46% and 20 men - RR 35.71%). Patient mean age was 36.5 years (SD 4.6). The majority of the patients did not have children at the time of the survey (n=96, 90.6%); a few had children at that time (n=10, 9.4%); and two (2%) were pregnant. Eighty-six patients (79.6%) were under treatment using their own gametes; 11 (10.2%) were under treatment using donated gametes; and ten (9.3%) were undergoing treatment for the first time.

### Somatic and psychological effects of ART treatments

The main somatic effects for patients undergoing ART treatments were swelling (39.8%), pain (12%), tiredness, and sensitivity (9.3% each). This is how some of the patients described their sensations and impressions: “*I was swollen and retaining water*”, “*My abdomen hurt*”, “*The hormones made me feel very tired, and the fact that I could not exercise most of the time impacted my self-esteem and mood*”, “*The treatment made me feel a little unwell*”.

In terms of psychological effects, patients reported anxiety (36.1%) and depression (30.6%) as the main impacts of infertility treatment. Guilt and hope (17.6% each) followed by powerlessness and shame (10.2% each) completed the list of top concerns. This is how some of the patients described their feelings: “*I have become more anxious and have had more trouble sleeping since I became more worried about aging*”, “*I think fear and frustration are pushing me into a state of depression. I wonder if a doctor might help me*”, “*I feel mostly sad and guilty, though I rationally know that low ovarian reserve is not tied to my behavior and that I should not blame myself for not being pregnant yet*", “*I hope I will get pregnant with a donor egg and become a mother*”, “*I feel powerless about a number of things, since I have not really discovered the cause of my infertility yet*”, “*I feel I am different because my friends are getting pregnant. I have backed off from some of them*”.

Effects of behavior and practical aspects were also extracted from the survey. Social relationships were more significantly affected (17.6%), as observed in the following example: “*In order to avoid questions, I have stopped getting along with people and I no longer attend events organized by family or friends*”. Time (47.2%) and financial constraints (13.9%) were the main practical difficulties of undergoing infertility treatment. In the words of the patients, “*The routine ultrasound scans affect my work*”, “*For at least three years I have changed my vacations or have not traveled at all because of the treatment*”, “*The financial factor is very prominent for us, mainly because we have tried it a few times and have not been successful yet*”.

### The top 30 research priorities

Table 1 lists the top 30 research priorities from the perspective of patients.

**Table 1:** Top 30 research priorities in infertility according to Brazilian patients.

TOPIC	FREQUENCY
1) Risks associated with ART	57.4%
2) How to cope with infertility (general)	56.5%
3) Success rates in ART	56.5%
4) Impact of diet in ART and fertility	47.2%
5) Long-term side effects of ART treatment	36.1%
6) Alternative therapies	29.6%
7) Impact of exercise on fertility and ART success	24.1%
8) Healthy habits (general)	31.5%
9) Side effects of ART treatments (general)	23.1%
10) Individual psychological support	22.2%
11) Oocyte quality and ovarian reserve	19.4%
12) Safety of ART treatments (general)	20.4%
13) Genetic or hereditary causes of infertility	18.5%
14) Impact of nervousness, stress and anxiety on treatment and fertility	15.7%
15) Effects of not taking contraceptive drugs on ART and fertility	13.0%
16) Implantation failure and miscarriage	13.0%
17) Endometriosis	13.0%
18) Male factor	13.0%
19) Available diagnostic tests for infertility	13.0%
20) Fertility preservation	13.0%
21) Psychological support (general)	13.0%
22) Impact of female age	12.0%
23) Idiopathic infertility	12.0%
24) Impact of being free from sexually transmitted diseases on infertility and ART outcomes	11.1%
25) Cancer risk due to ART treatment	10.20%
26) How to cope with a negative result	9.3%
27) Composition and mode of action of ART drugs	9.3%
28) Cost of treatment	8.3%
29) Tailored treatments and new techniques under research	6.5%
30) Psychological support group	6.5%

Patients generally picked research topics related to lifestyle habits (impact of diet, exercises, and alternative therapies including yoga, meditation, and acupuncture), risks, safety, side effects of ART treatment, psychological aspects (how to cope with infertility and the need for psychological support), treatment success rates, and infertility causes (oocyte quality and ovarian reserve, genetic or hereditary causes, among others).

The most cited concern covered the risks associated with ART. This is how some of the patients voiced their interests in the matter: *"Can treatment cause genetic changes in the baby or make them more susceptible to diseases?"*, “*Is there a greater risk of congenital malformation that is not related to chromosomal disorders?"*.

Surprisingly, patients placed the importance of psychological aspects above ART treatment success rates. These are some of the questions raised on the subject: *"Can emotional factors disrupt fertility?"*, *"How much can emotional factors interfere with the outcome of treatment?"*, *"What is the best therapy in psychology to help cope with the issues of infertility?"*.

ART treatment success rate ranked third in the list of research priorities in our study. Here is what some of the patients said about it: *"What is the actual treatment success rate?"*, *“A lot of people suffer from this condition and seek treatment for it. Since everything is registered, I wonder if it might be possible to use these data to generate patient profiles and assign them chances of success and failure, in a way that we are more comfortable with knowing the group we belong to".*

Patients mentioned lifestyle aspects and their role in fertility and ART treatment outcomes. Food, exercise, and alternative therapies were mentioned, as shown in the following patient remarks: *"Is there a lifestyle that is less conducive to infertility?", "Are there foods or vitamins that might help with infertility prevention?"*, *“Do pineapples and coconut water actually help the embryo in the uterus?"*, *“What kind of physical exercises, if any, are contraindicated for infertile people?"*.

Treatment side effects appeared in the top 30 research priorities under two topics: long-term side effects of treatment and treatment side effects in general. Patients cited the effects of ART treatment drugs - and more specifically the chances of developing a secondary disease such as cancer. Patient remarks included: “*What is the effect on health of undergoing several cycles?*" and “*I would like to know the actual risk of having cancer because of the treatment, and if individuals with a family history of cancer are more likely to have the disease if they undergo IVF*”. Concerns over short-term side effects included the immediate side effects of medication on patient mood: “*I wonder if the medication used during treatment affects my mood. I feel that I have become more emotional and less patient, possibly because of the anxiety generated by the treatment. Is there evidence linking mood swings to ART treatment medication?*” In terms of the general safety of ART treatment, patients wondered whether the use of other drugs might affect treatment efficacy: “*Can medications such as anti-inflammatory and antibiotic agents affect my treatment?*", “*Does levothyroxine interfere with treatment at all?*”.

The included patients also mentioned causes of infertility. The most frequent topic was oocyte quality and ovarian reserve: *"I would like to learn more about the biological mechanisms related to infertility in young women with low ovarian reserve."* Genetic and hereditary causes of infertility were also cited: *"Is Infertility a condition that you develop or is it already defined in your DNA?"* Other cited causes included implantation failure and miscarriage (*"Why does implantation failure happen?"*), endometriosis (*"Even if they have had surgery for endometriosis, why do many people still have trouble getting pregnant?"*), and male factors (*"Is there a way to prevent sperm DNA fragmentation?”*). Other subjects cited by patients were the impact of female age and idiopathic infertility.

### The top 10 research priorities

Table 2 lists the top 10 research priorities extracted from top 30 research priorities identified by the surveyed patients. Closely related categories were grouped into a summarized list.

**Table 2:** Top 10 research priorities in infertility according to Brazilian patients.

TOPIC	FREQUENCY
1) Short- and long-term side effects of ART treatments (general safety)	83.3%
2) How to cope with infertility (general and specific situations)	80.6%
3) Risks associated with ART	57.4%
4) Success rates in ART	56.5%
5) Impact of diet on ART and fertility	47.2%
6) Healthy habits (general)	31.5%
7) Alternative therapies	29.6%
8) Impact of exercise on fertility and ART success	24.1%
9) Oocyte quality and ovarian reserve	19.4%
10) Genetic or hereditary causes of infertility	18.5%

The most frequent research priority category cited by Brazilian patients was side effects of ART treatment (83.3% frequency) - which comprised general, short- and long-term side effects and the overall safety of ART treatments - followed closely by how to cope with infertility (80.6%). The risks associated with ART (57.4%) and treatment success rates (56.5%) ranked third and fourth, respectively. The fifth to eighth priorities were all related to lifestyle habits: impact of diet on ART and fertility (47.2%), general healthy habits (31.5%), alternative therapies (29.6%), and impact of physical exercises on fertility and ART success (24.1%). The last two research priorities in the top 10 list featured causes of infertility: oocyte quality and ovarian reserve (19.4%) and genetic or hereditary causes (18.5%).

## DISCUSSION

This study identified the main research interests of Brazilian patients in the field of infertility and ART treatments. A long list of 30 and a shortlist of 10 top research topics were extracted from a validated JLA survey model. Short- and long-term side effects, psychological aspects, and risks associated with infertility treatment were the main topics of research interest raised by patients.

The top two research priorities for patients were very close in frequency (83.3% and 80.6%). General safety and side effects of ART treatment (83.3%) was cited as a priority for research efforts from the perspective of patients. Questions regarding the effects of hormone therapy, the pharmacological interactions between drugs for chronic disease and IVF treatment, the effects of treatment on patient mood, and the chances of developing cancer as a secondary effect of ART treatment were the main topics raised by the surveyed patients. Researchers, clinicians, and the pharmaceutical industry have continuously attempted to improve ART treatments and enhance the chances of patients achieving healthy pregnancies. Changes introduced in ovarian stimulation over time have resulted in less aggressive protocols, particularly since the advent of molecular biology and the production of recombinant gonadotropins, which have provided for more efficient and safer ART cycles (Santos-Ribeiro *et al.,* 2019). However, it is important to stress that adverse events may still occur, most of which are related to ovary stimulation. Long-term side effects of ovary stimulation drugs are debated by researchers due the difficulty in establishing a border between causes of female infertility (such as anovulation and endometriosis) and use of ART drugs and its association with cardiovascular diseases and cancer (Santos-Ribeiro *et al.,* 2019).

Psychological issues were mentioned by 80.6% of patients. In terms of short-term side effects, patients wondered whether ART drugs affected their emotional health. Most patients raised the need for psychological support to deal with infertility, especially when it came to coping with situations they faced during ART treatment. Facing infertility is, per se, a distress factor that affects quality of life and social interactions (Massarotti *et al.,* 2019). ART treatment is demanding, especially for the female partner, who must undergo ovarian stimulation, egg retrieval, and embryo transfer procedures. Unsurprisingly, women are more psychologically affected by ART treatment and often present symptoms of anxiety and depression (Massarotti *et al.,* 2019). Psychological support is a key element in patient wellbeing and should be a priority for clinicians during counseling (Massarotti *et al.,* 2019). In line with this topic, patients wondered if alternative therapies might help them manage anxiety and alleviate the stress caused by ART treatment. Acupuncture, yoga, and meditation were cited as the main alternative therapies. In a systematic review, LoGiudice & Massaro (2018) discussed the impact of mind-body techniques (mainly Hatha yoga, cognitive behavioral interventions, and mind-body therapies) on women undergoing IVF. The authors showed a clear benefit in terms of decreased anxiety and stress levels for IVF patients on alternative therapies, although the impact of these therapies at improving reproductive outcomes is still debatable. However, female patients under less stress are less likely to give up ART therapy, which possibly increases their chances of achieving successful reproductive outcomes (LoGiudice & Massaro, 2018). In a systematic review and meta-analysis on the use of acupuncture in IVF treatment, Qian *et al*. (2017) reported improvements in clinical pregnancy rates. However, another systematic review and meta-analysis by Smith *et al*. (2019) on acupuncture performed around the day of embryo transfer found no evidence of increased pregnancy rates resulting from the procedure. In summary, the ability of alternative therapies to improve reproductive outcomes is still being discussed, although there is consensus around the improvements in quality of life and decreased levels of distress and anxiety in ART patients (LoGiudice & Massaro, 2018).

Another theme of research interest indicated by the patients was the effect of diet, exercises, and healthy habits in fertility and ART treatment. It is well known in the scientific community that lifestyle habits have a direct impact on fertility (Sharma *et al.,* 2013). Women who consume a higher monounsaturated to trans-fat ratio, high-fat over low-fat dairy products, plant over animal protein, decreased glycemic load and increased intake of multivitamins and iron have lower rates of ovulatory disorders and infertility (Chavarro *et al.,* 2007). Men with diets rich in fiber, lycopene, folate, fruit, and vegetables have improved semen quality (Sharma *et al.,* 2013). Overweight (body mass index - BMI - between 25 and 30) and obesity (BMI above 30) have been correlated with ovulatory dysfunction, miscarriage, and lower implantation rates in women, and with decreases in sperm motility and concentration, and increased DNA damage in men (Sharma *et al.,* 2013). Malnutrition may also affect fertility in both men and women, although research in this field is scarcer than on obesity. In women, malnutrition may affect ovulatory physiology and menstrual cycles due to low body fat, while lower semen concentrations can occur in males (Sharma *et al.,* 2013). Exercise is beneficial for fertility parameters in men and women when practiced with moderation - at least three times a week for one hour (Sharma *et al.,* 2013). Moreover, it provides a short-term benefit for fertility especially during the period that covers preconception, including the maturation of gametes and early embryo development, which are affected by parental physiology, body composition, metabolism, and diet. Thus, exercises affect the developmental programming of offspring and risks of long-term comorbidities and chronic diseases in adulthood such as immune, metabolic, and neurological morbidities and cardiovascular disease (Fleming *et al.,* 2018). However, changes in habits are entirely dependent on individual efforts; thus, clinicians and caregivers should advise their patients on healthy lifestyle counseling (Sharma *et al.,* 2013).

The included patients also mentioned the risks associated with ART. The main concern revolved around the potential implications of ART on the health of their offspring. The health of children born from IVF procedures - more than seven million individuals today - has been investigated in large birth cohort studies by matching them against their spontaneously conceived peers. In regard to the risks of ART procedures, intracytoplasmic sperm injection (ICSI) increases the risk of poor semen quality in the offspring, with no correlation to the semen quality of their fathers (Belva *et al.,* 2016). ICSI has also been associated with birth defects (Berntsen *et al.,* 2019); however, it provides for improved obstetric and perinatal outcomes - preterm birth - compared with babies born after IVF (Berntsen *et al.,* 2019). Adverse obstetric and perinatal outcomes of ART-conceived pregnancies have been extensively reported (Berntsen *et al.,* 2019). Small for gestational age (SGA) neonates, preterm birth, and low birth weight have been associated with fresh embryo transfers, while pre-eclampsia, other hypertensive disorders, and large for gestational age (LGA) neonates have been associated with frozen embryo transfers. Questions regarding diseases that are not associated with chromosomal anomalies - the likes of autism, cancer, and birth defects - have also been investigated in large cohort studies. Autism spectrum disease (ASD) has been associated with multiple pregnancies from ART (Liu *et al.,* 2017), while children and young adults (aged 21 years) born from ART are not at increased risk of cancer (Spaan *et al.,* 2018, Berntsen *et al.,* 2019). The risk of cancer in adulthood of individuals born from ART has not been discussed in the literature. The incidence of congenital birth defects is higher in ART babies, the most common of which being cardiovascular defects (Berntsen *et al.,* 2019). ART treatments have not been around for long. Therefore, in order to assess the consequences of ART on the health of children and adults born from IVF, it is crucial that the healthcare and scientific communities implement efforts to follow these individuals throughout their lives and develop strategies to diminish potential negative effects arising from the use of ART.

Patients also indicated that causes of infertility should be assigned higher priority in research efforts. The preferred topics were oocyte quality, ovarian reserve, and genetic/hereditary causes of infertility. Ovarian dysfunction is present in 25% of women with infertility (National Collaborating Centre for Women’s and Children’s Health, 2013). Due to postponement of childbearing, low ovarian reserve has become an important matter in female infertility (Ubaldi *et al.,* 2019). The growing number of fertility preservation cycles performed for social reasons in IVF clinics in developed countries signals the success of the efforts made to communicate the effects of female age on oocyte quality and ovarian reserve (Alteri *et al.,* 2019). However, a significant portion of the population still cannot afford ART procedures. Patients are also concerned about the role of genetics and inheritance in infertility, especially whether infertility is coded in the DNA or is acquired during life. Certain gene polymorphisms have been correlated with infertility in men and women (Casarini *et al.,* 2015). Genetic factors and epigenetic alterations associated with infertility and ART may be transmitted to the offspring of infertile couples (Berntsen *et al.,* 2019). However, research involving human subjects on this topic is scarce. The majority of the studies published in the literature are based on animal models (Berntsen *et al.,* 2019).

Although the scientific community has addressed some of the research priorities listed above, there is an important lack of communication between healthcare providers and patients. As important as research results *per se* is the translation of research findings for the general population so that they are better equipped to balance the risks and benefits of ART treatments and choose methods to improve quality of life is a must.

Our study had its limitations, some of which are discussed herein. The participants included in our study attended private fertility centers. Therefore, their research preferences and priorities may differ from those of individuals seen at public fertility clinics. Female patients accounted for the majority of survey respondents. Therefore, the research priorities and preferences of male patients may have been underrepresented.

## CONCLUSIONS

Patient research priorities may differ from the priorities of clinicians, researchers, and healthcare providers in many areas of medicine (Crowe *et al.,* 2015). To better cater to the needs of patients and develop patient-centered care in the field of infertility and ART treatments, clinicians, healthcare providers, and the scientific community must have a clearer understanding of their patients’ concerns and make efforts to address them properly.

## Annex 1 Survey Questions

**You are a:**

-Male
-Female

**In which city do you live?**

-São Paulo, SP
-Greater São Paulo (Santo André, São Bernardo, São Caetano, Diadema, Osasco)
-Campinas
-Other city in the State of São Paulo
-Other Brazil State (please indicate the city and state):
-Other country (please indicate the city and country):

**How old are you?**

**Do you have children?**

Yes

No

**Which of the following best describes you? Please check all that apply**

I (or my partner) cannot get pregnant naturally

I have had infertility treatment in the past

I will probably have infertility treatment in the future

My gametes (eggs or sperm) were used in my treatment

The gametes of a donor (eggs or sperm) were used in my treatment

**How does infertility affect your daily life? Please tell us about physical, emotional and social effects.**

Examples: Do you feel different from other people? Do you experience pain that prevents you from doing certain things? Did you change your social interactions because of infertility?

**How do the treatments that you receive affect your daily life? Please tell us about physical, emotional and social effects.**

Examples: Does hormonal stimulation make you feel bloated? Do injections disrupt your work schedule? Did you change vacation plans because of treatment? Do you find it difficult to see your doctor for treatment?

There are many things that we do not know about infertility. We would like to hear from you what matters to you, so that we can realign our research focus and efforts.

What relevant topics do you believe research should look into? Please see the examples below and think about your own questions. Let us know the areas in which you think more investigation is needed in the space provided below.

**1. Do you have any questions about what causes infertility?**

Example: A similar survey in people with kidney disease identified that they wanted to know if certain errors in DNA might cause renal disease.

**2. Do you have any questions about what can be done to prevent infertility?**

**3. Do you have any questions about the day-to-day life of people living with infertility?**

Example: In a similar survey, people with depression wanted to know how to best identify a bout of depression before it happened*.*

**4. Do you have any questions about the medication used for fertility treatment?**

Example: In a similar survey, people with diabetes asked which kind of insulin was safest and had the fewest side effects.

**5. Do you have any questions about the emotional aspects of infertility and fertility treatments?**

Example: People who had cancer wanted to know whether personalized psychological support might improve their recovery.

**6. Do you have any questions about the outcomes of infertility treatment?**

Example: People who break a leg might want to know whether surgery might allow them to go back to walking faster than putting a cast on.

**7. Do you have any questions about the safety of infertility treatment for the mother and the conceived child?**

**8. Do you have any questions about other aspects of treatment (diet, exercise or alternative medicine) for infertility?**

Example: Individuals who had muscle injuries wanted to know whether acupuncture might accelerate their recovery.

**9. Do you have any questions that you feel are important for researchers to answer but do not fall into the areas specified above?**

Example: Individuals who had a stroke wanted to know whether stem cell research might be beneficial for them.
